# The Endothelial Glycocalyx and Retinal Hemodynamics

**DOI:** 10.3390/pathophysiology29040052

**Published:** 2022-12-17

**Authors:** Gaganpreet Kaur, Wendy Leskova, Norman R. Harris

**Affiliations:** Department of Molecular and Cellular Physiology, Louisiana State University Health Sciences Center at Shreveport, 1501 Kings Highway, Shreveport, LA 71103, USA

**Keywords:** endothelial glycocalyx, retinal hemodynamics, heparinase, hyaluronidase, microhematocrit

## Abstract

Purpose. Previous studies suggest that the endothelial glycocalyx adds to vascular resistance, inhibits thrombosis, and is critical for regulating homogeneous blood flow and ensuring uniform red blood cell (RBC) distribution. However, these functions and consequences of the glycocalyx have not been examined in the retina. We hypothesize that the endothelial glycocalyx is a critical regulator of retinal hemodynamics and perfusion and decreases the propensity for retinal thrombus formation. Methods. Hyaluronidase and heparinase, which are endothelial glycocalyx-degrading enzymes, were infused into mice. Fluorescein isothiocyanate–dextran (2000 kDa) was injected to measure lumen diameter, while RBC velocity and distribution were measured using fluorescently labeled RBCs. The diameters and velocities were used to calculate retinal blood flow and shear rates. Mean circulation time was calculated by measuring the difference between arteriolar and venular mean transit times. Rose Bengal dye was infused, followed by illumination with a green light to induce thrombosis. Results. The acute infusion of hyaluronidase and heparinase led to significant increases in both arteriolar (7%) and venular (16%) diameters in the retina, with a tendency towards increased arteriolar velocity. In addition, the degradation caused a significant decrease in the venular shear rate (14%). The enzyme infusion resulted in substantial increases in total retinal blood flow (26%) and retinal microhematocrit but no changes in the mean circulation time through the retina. We also observed an enhanced propensity for retinal thrombus formation with the removal of the glycocalyx. Conclusions. Our data suggest that acute degradation of the glycocalyx can cause significant changes in retinal hemodynamics, with increases in vessel diameter, blood flow, microhematocrit, pro-thrombotic conditions, and decreases in venular shear rate.

## 1. Introduction

The endothelial glycocalyx (EG) is a carbohydrate-rich dynamic layer present on the apical surface of the vascular endothelium. The primary backbone molecules of the EG are proteoglycans comprised of core proteins and glycosaminoglycans (GAGs). The core proteins are either transmembrane proteins from the syndecan family (syndecan-1,2,3,4) or glycosylphosphatidylinositol-anchored glypican-1 [1]. Heparan sulfate (HS), chondroitin sulfate (CS), and hyaluronic acid (HA) are the major GAGs forming the mesh-like network of the glycocalyx. HS and CS bind covalently to syndecans and glypicans, whereas HA binds to its receptor CD44 and RHAMM [1].

The EG is critical for the maintenance of vascular endothelial homeostasis. The presence of sulfated molecules provides a negative charge to the glycocalyx; therefore, it negatively repels charged molecules and blood cells and contributes to a “cell exclusion zone”. Furthermore, the endothelial glycocalyx acts as a molecular sieve for plasma proteins, including albumin, which gets adsorbed on the glycocalyx layer, contributes to intravascular osmotic pressure, and creates an oncotic pressure gradient between the lumen and subglycocalyx space. Therefore, the EG regulates fluid movement between the lumen and interstitial space [2,3]. In addition, the presence of EG components, possibly heparan sulfate and syndecan-4, aligns endothelial cells in the direction of flow [4,5] and modifies the level of fluid mechanical shear stress on endothelial cells [2,6]. The EG also plays a critical role in shear-induced nitric oxide (NO) production, a major vasodilator. Shear stress is sensed by HS and glypican-1, which initiates the phosphorylation of platelet endothelial cell adhesion molecule-1 that results in the phosphorylation of endothelial nitric oxide synthase and thereby NO production [2,7]. Therefore, the endothelial glycocalyx is a major determinant of vascular physiology and health.

The endothelial glycocalyx is in continuous contact with the blood; therefore, its contributions to flow resistance are recognized. However, there are conflicting reports concerning the role of the EG in blood flow regulation, with a need for further clarification. In a previous study, the enzymatic removal of HS using heparinase resulted in a 14–21% decrease in resistance (equivalent to a ~1 μm increase in vessel diameter) in the arterioles of rat mesentery [8]. However, the infusion of hyaluronidase, which degrades HA, significantly reduced arterial conductance and increased flow resistance in the iliac of anesthetized pigs [9]. Therefore, it is possible that individual components of the EG regulates blood flow differently and may have tissue-specific roles. To our knowledge, no study has investigated the role of the EG in the regulation of retinal hemodynamics and perfusion.

Although not yet determined in the retina, it has been demonstrated that the EG mediates red blood cell (RBC) distribution. The degradation of the EG using light-dye treatment, or with heparinase or hyaluronidase, resulted in a 60–100% increase in capillary hematocrit [10,11,12]. In addition, an empirical computational study suggested that the presence of the EG is critical for the homogeneous composition of the blood and the uniform distribution of circulating RBCs [13]. These findings were further supported by an experimental study where the authors demonstrated altered capillary perfusion following the enzymatic removal of HA, which can impair oxygen transport and cause regional tissue hypoxia. The hyaluronidase treatment decreased functional capillary density (FCD), defined by the presence of at least one RBC in the vessel. The reduction in FCD was accompanied by an increase in no-flow capillaries, plasma-only perfused capillaries, and an increase in capillary hematocrit in the remaining functional capillaries [14].

The EG is a well-established regulator of vascular homeostasis. However, the functions of the EG in the retina have not been well studied. The impairment of the retinal endothelial glycocalyx [15,16,17,18,19,20] and retinal hemodynamics [21,22,23,24,25] has been observed in several diseases, including diabetes and hypertension; therefore, it is important to investigate the link between the two. Our hypothesis is that the endothelial glycocalyx is a critical regulator of retinal hemodynamics and perfusion and adds to flow resistance. We also tested a hypothesized effect of the loss of the glycocalyx on increased retinal hematocrit and the increased propensity for thrombus formation.

## 2. Materials and Methods

### 2.1. Animals

C57BL/6J male mice were obtained from The Jackson Laboratory (Bar Harbor, ME, USA) and housed until 3–4 months of age, with ad libitum access to food and water with a 12 h light/dark cycle. Animal protocols were approved by the Institutional Animal Care and Use Committee of Louisiana State University Health Sciences Center-Shreveport. All experiments were designed and carried out in accordance with the ARVO Statement for the Use of Animals in Ophthalmic and Vision Research.

### 2.2. Enzyme Treatments

The endothelial glycocalyx was degraded by the infusion of hyaluronidase from bovine testes (25 mg/kg/body weight; H3884, Sigma, St. Louis, MO, USA) and a blend of heparinase I and III from *Flavobacterium heparinum* (1 U/mouse; H3917, Sigma, St. Louis, MO, USA) for 30 min. Heparinase units are given as Sigma units. The enzymes were prepared in phosphate-buffered saline (PBS) and were infused through femoral vein cannulation. The control animals were infused with PBS alone for 30 min. We have successfully demonstrated in a previous study a significant degradation of the mouse retinal glycocalyx following hyaluronidase infusion at a dose of 12 mg/kg [17].

### 2.3. Preparative Surgery for Intravital Microscopy

The mice were anesthetized intraperitoneally with a cocktail of ketamine (120 mg/kg) and xylazine (16 mg/kg) and were kept on a heating pad for the duration of the experiment. Both eyes were moistened with PBS during the cannulation and enzymatic treatment procedures. The lower abdomen was shaved, followed by an incision, and the femoral vein was cannulated with polyethylene tubing (PE10). Prior to the placement on the intravital microscope, the mouse was placed on its right side with gauze underneath the head for support, and the pupil of the left eye was dilated using Tropicamide Ophthalmic Solution USP 1% (Falcon Pharmaceuticals Ltd.; Fort Worth, TX, USA). Following dilation, one drop of Gonak Hypromellose Ophthalmic Demulcent Solution, 2.5% (Akron Inc.; Buffalo Grove, IL, USA) was placed topically on the eye, which was then covered with a 5 mm circular glass coverslip to allow visualization of the retinal vasculature.

### 2.4. Measurements of Retinal Velocity, Diameter, and Hematocrit

RBCs were fluorescently labeled as described previously in [26,27]. Whole blood was collected from age-matched donor mice in a 1 mL syringe filled with 100 µL anticoagulant citrate dextrose (Sigma, St. Louis, MO, USA). Following the collection, whole blood was passed through a 5 mL syringe containing 1 g of cotton, and the syringe was further eluted with 10 mL PBS. The blood solution was spun down for 10 min at 3000 rpm, the supernatant removed, and the pellet resuspended in 1 mL PBS. The RBC pellet was centrifuged (3000 rpm at 5 min) again and washed twice with PBS. The RBCs were fluorescently labeled with 1,1′-dioctadecyl-3,3,3′,3′-tetramethyl-indocarbocyanine perchlorate (DiI, Invitrogen Molecular Probes, Eugene, OR, USA) and washed five times with PBS. After washing, 100 µL DiI-labeled RBC were resuspended in 1.2 mL PBS and used the same day or stored at 4 °C overnight. Prior to infusion, labeled RBCs were centrifuged (3000 rpm for 5 min) and washed in PBS thrice.

Each mouse was infused with 1.8 mL/kg labeled RBCs, which is approximately 10% of the total number of circulating RBCs in mice. After 5 min of infusion, images were captured using a Nikon Eclipse E600FN microscope through a 10× objective and rhodamine filter (excitation wavelength of 540–580 nm and emission wavelength of 600–660 nm). Images were captured using an exposure time of 5 ms. DiI-labeled RBCs appeared as fluorescent streaks in the vessels, where the length of the streak was proportional to the velocity of the RBC. Images were analyzed using NIS Elements Basic Research software version 2.1–2.3 (Nikon Instruments Inc., Melville, NY, USA). Ten sequential RBC streaks were averaged per vessel; the mean velocity for arterioles and venules is reported in mm/s.

**Figure 1:**
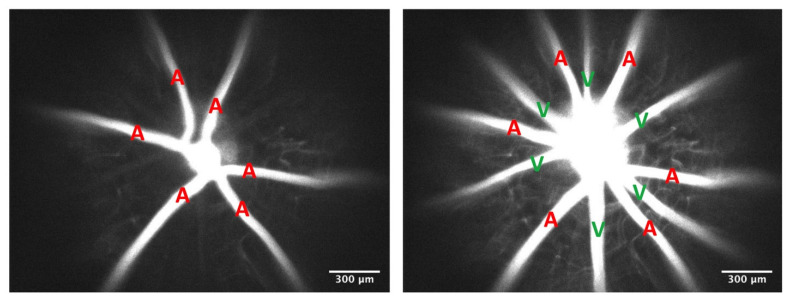
Filling of FITC–dextran in the Retinal Circulation following Infusion. The vessels filling first with infused FITC–dextran are arterioles (A) and the vessels filling later are venules (V). Scale bar = 300 µm.

To verify whether the vessel was an arteriole or a venule, a 50 µL bolus of fluorescein isothiocyanate (FITC)–dextran (2,000,000 MW, 4.5 mg/kg; Sigma, St. Louis, MO, USA) was injected through the femoral vein. The vessels filling first with FITC–dextran were considered to be arterioles, and the vessels filling later were considered to be venules (Figure 1). Videos of FITC–dextran filling the retinal circulation were recorded with a Photometrics CoolSnap ES camera at 4× magnification, followed by consecutive focusing on the arterioles and venules for diameter measurements using 10× magnification. The diameter of each vessel was measured at 5 different locations/vessel at least 30 µm apart and within 600 µm of the optic disc, and an average of the five measurements/vessel were then averaged together for the reported arteriolar and venular diameters for a given mouse. Diameters were examined using ImageJ software (Version 1.53a; National Institutes of Health; Bethesda, MD, USA).

To inspect RBC distribution in the retina, cells labeled with DiI and FITC–dextran were injected (as described previously) in two separate sets of animals. After 5 min of infusion, the eyes were excised, and the retinas were enucleated for retinal flat mounts. These flat mounts were imaged using a Nikon Eclipse E600FN microscope with 10× magnification and both FITC and rhodamine filters. The number of RBCs per 10× field of view was counted for the retinal vasculature.

### 2.5. Measurements of Retinal Flow Rate and Shear Rate

Vascular blood flow was calculated as velocity × cross-sectional area (= π × diameter^2^ ÷ 4) for individual arterioles and venules, which were summed to calculate total arteriolar and venular blood flow, respectively. The total flow in arterioles and venules was averaged for a calculation of the total retinal blood flow. The shear rate of individual arterioles and venules was calculated as 8 × velocity/diameter and averaged to calculate the mean arteriolar and venular shear rate.

### 2.6. Measurements of Mean Circulation Time

As described previously, the mean transit time was calculated based on the fluorescent intensity curves of the infused FITC–dextran bolus through the arterioles and venules [24,27]. The fluorescent intensities were measured in the middle of each vessel at a distance of 300 µm from the optic disk. Intensities were quantified in each frame, starting from 3 to 4 frames prior to the appearance of dye in the arterioles until the intensities in the arterioles and venules were affected by recirculation from the heart, and curves from individual arterioles and venules were averaged to determine the intensity profiles with respect to time. The recirculating FITC–dextran returning from the heart obscures the end of the intensity curves; thus, a logarithmic fit of the declining phase of the curve was extrapolated from the initiation of the recirculation, as shown previously [24]. The difference in arteriolar and venular mean transit time was calculated as mean circulation time (MCT).

### 2.7. In Vivo Thrombosis Induction

Carboxyfluorescein succinimidyl ester (CFSE, 25 µg/mouse, Enzo, Life Sciences; Farmingdale, NY, USA) was injected intravenously (to label leukocytes and platelets) after the retinal vessels of the left eye were brought into focus under 4× magnification using a fluorescein filter on a Nikon Eclipse E600FN microscope, with videos captured using a Sony 3CCD ExwaveHAD camera. After switching the microscope to a dual fluorescein/rhodamine filter, based on a previously established technique [28,29], thrombosis was induced by the infusion of rose Bengal dye (18 mg/kg; Sigma) and illumination with green light. The videos were analyzed for 16 min after the infusion of rose Bengal (or until all vessels stopped flowing). The time required for thrombosis initiation, the ratio of vessels affected, and the ratio of vessels with stopped-flow were recorded.

### 2.8. Statistics

Data are presented as means with 95% confidence intervals (CI), and *p* < 0.05 was considered statistically significant. The normality of data was tested using the Shapiro–Wilk test or Kolmogorov–Smirnov test. Data passing the normality test were analyzed using an unpaired, two-tailed Student’s *t*-test, while a Mann–Whitney U test was used to analyze data failing the normality test. All statistical analyses were performed using GraphPad Prism 9 software (version 9.1.1).

## 3. Results

### 3.1. Effect of the Endothelial Glycocalyx on Retinal Hemodynamics

**Figure 2:**
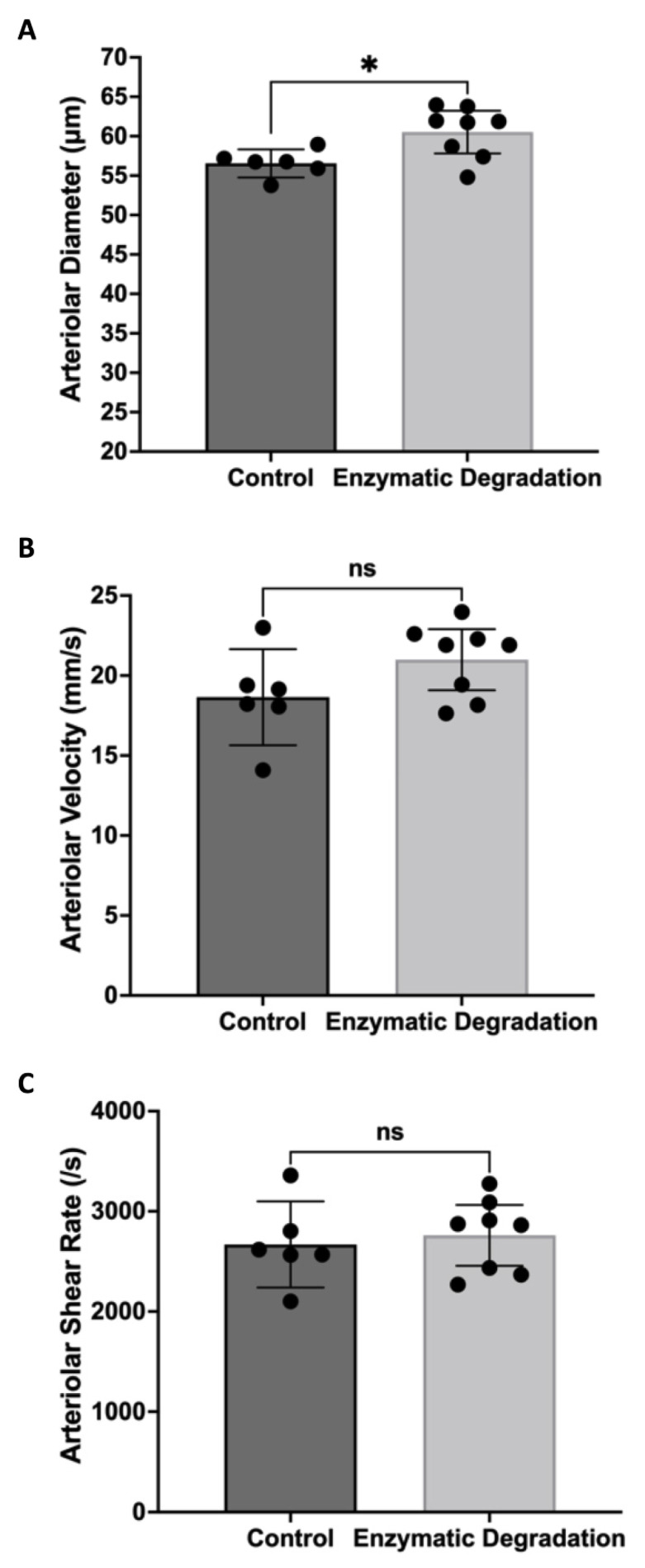
Retinal Arteriolar Hemodynamics. Retinal arteriolar diameter (**A**), velocity (**B**), and shear rate (**C**) in control mice (n = 6) and mice infused with glycocalyx-degrading enzymes (n = 8); means with 95% confidence intervals. * *p* < 0.05 and ns = nonsignificant.

**Figure 3:**
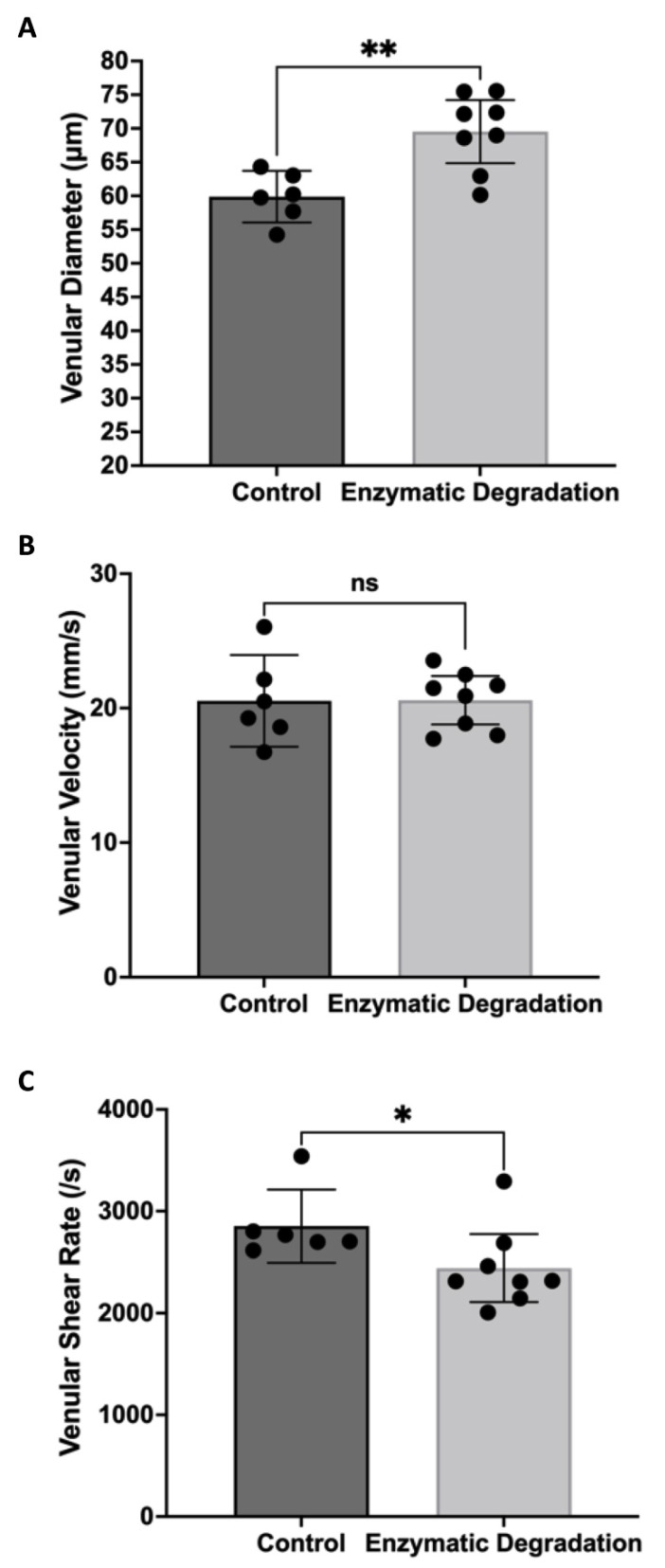
Retinal Venular Hemodynamics. Retinal venular diameter (**A**), velocity (**B**), and shear rate (**C**) in control mice (n = 6) and mice infused with glycocalyx-degrading enzymes (n = 8); means with 95% confidence intervals. * *p* < 0.05, ** *p* < 0.01, and ns = nonsignificant.

Figure 1 shows the filling of FITC–dextran in the retinal circulation following infusion. The vessels filling first with FITC–dextran are arterioles, and the vessels filling later are venules. Infusion of heparanase and hyaluronidase caused significant increases in arteriolar and venular lumen diameters filled with high molecular weight FITC–dextran, with a more pronounced effect on venules. Animals infused with the enzymes exhibited 7% greater arteriolar diameters (60.5 ± 2.7 µm vs. 56.6 ± 1.8 µm, *p* < 0.05, Figure 2A) and 16% greater venular diameters (69.5 ± 4.7 µm vs. 59.8 ± 3.7 µm, *p* < 0.01, Figure 3A) as compared to PBS-treated animals. We further observed a nonsignificant 10% increase in arteriolar velocity (18 ± 3.0 mm/s vs. 21 ± 1.9 mm/s, *p* = 0.11, Figure 2B) following infusion of the glycocalyx degrading enzymes. Venular velocity (~20 mm/s) was not altered in mice with enzymatic degradation of glycosaminoglycans compared to the control mice (*p* = 0.97, Figure 3B).

With the shear rate proportional to velocity/diameter, the significant increase in arteriolar diameter coupled with a slight (nonsignificant) increase in arteriolar velocity did not lead to any substantial changes in arteriolar shear rate (2669 ± 430 s^−1^ in control mice vs. 2760 ± 303 s^−1^ with enzymes, Figure 2C). However, the significant increase in venular diameter coupled with no change in venular velocity led to a significant decrease in venular shear rate in mice injected with hyaluronidase and heparinase compared to mice injected with PBS (14% decrease, 2854 ± 359 s^−1^ vs. 2442 ± 333 s^−1^, *p* < 0.05, Figure 3C).

### 3.2. Effect of the Endothelial Glycocalyx on the Retinal Blood Flow and MCT

**Figure 4:**
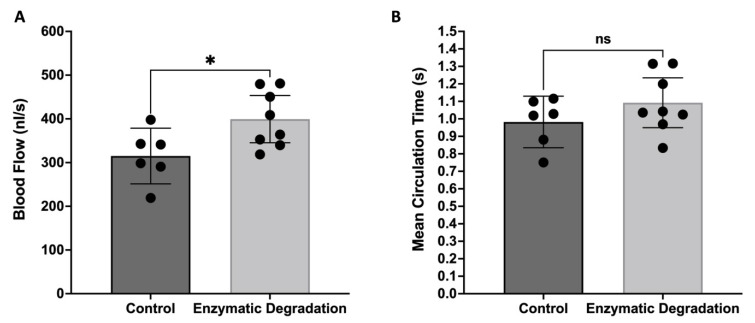
Effect of the Endothelial Glycocalyx on Retinal Blood Flow and MCT. (**A**) Total retinal blood flow and (**B**) mean circulation time through the retina in control mice (n = 6) and mice infused with glycocalyx-degrading enzymes (n = 8); means with 95% confidence intervals. * *p* < 0.05 and ns = nonsignificant.

The total retinal blood flow rate, measured using velocities and diameters, is presented in Figure 4A. Retinal flow increased from 315 ± 64 nL/s in control mice to 400 ± 54 nL/s in mice with the enzymatic degradation of glycosaminoglycans (27% increase, *p* < 0.05). Given the slight increase in arteriolar velocity, it might be expected that mean transit time through the retina (or mean circulation time, MCT) would decrease. However, enzymatic degradation of the endothelial glycocalyx caused a negligible change in MCT (0.98 ± 0.15 s in control mice vs. 1.09 ± 0.14 s in enzyme-infused mice, Figure 4B).

### 3.3. Effect of the Endothelial Glycocalyx on Retinal RBC Distribution

**Figure 5:**
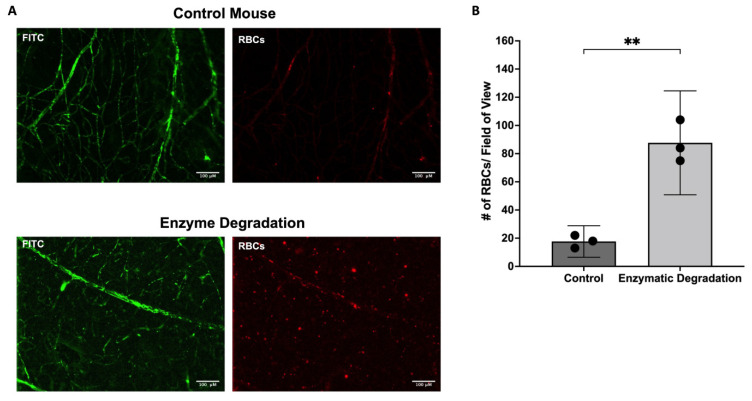
Effect of the Endothelial Glycocalyx on Retinal Microhematocrit. (**A**,**B**) Representative images and quantification of the number of RBC per field of view in control mice and mice infused with hyaluronidase and heparinase (n = 3); means with 95% confidence intervals. ** *p* < 0.01. Scale bar = 100 µm.

To examine the retinal distribution of RBC, flat mounts were randomly imaged in each of the four quadrants. RBC distribution was uniform among all quadrants in both the control and enzyme-infused retinas. However, the overall number of RBCs in the retina significantly increased with the degradation of the vascular glycocalyx (*p* < 0.01, Figure 5A,B). On average, ~18 RBCs were present per field of view (~0.60 mm^2^) in control mice compared to the ~88 cells in mice infused with hyaluronidase and heparinase.

### 3.4. Effect of the Endothelial Glycocalyx Degradation on Thrombosis Induction

**Figure 6:**
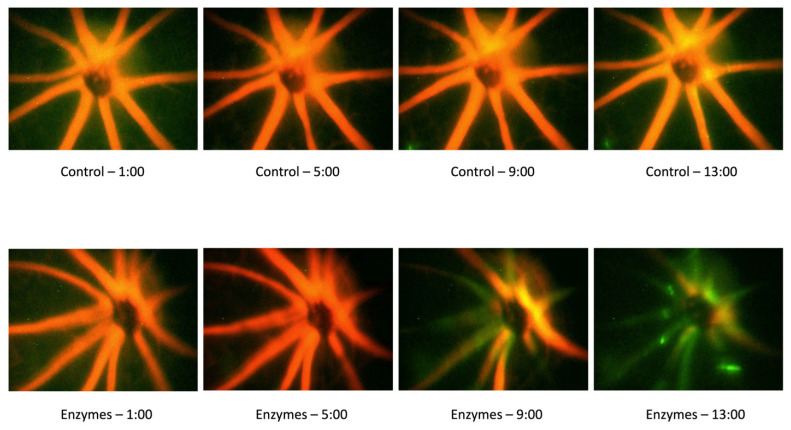
Representative Video Frames from the Thrombosis Experiments. Video frames of a control mouse and a mouse that had been infused with the GAG-degrading enzymes at times of approximately 1, 5, 9, and 13 min following the infusion of the Rose Bengal dye. The loss of red dye indicates the stoppage of flow due to the occlusion of the vessels.

**Figure 7:**
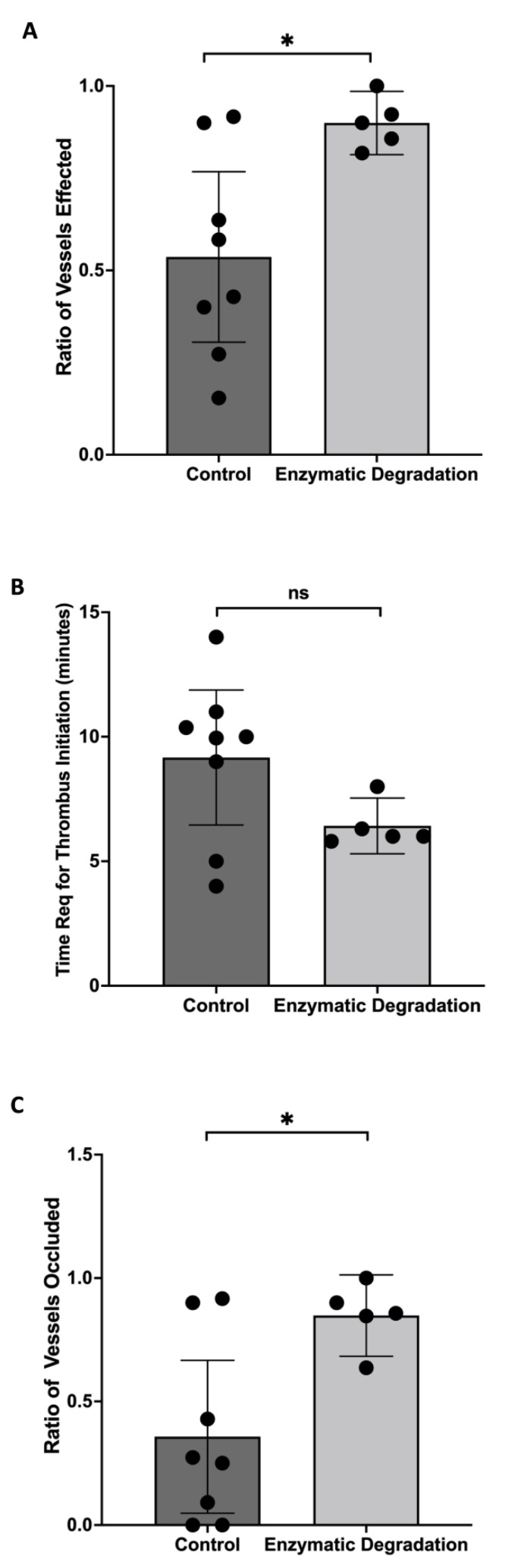
Effect of Endothelial Glycocalyx Degradation on Thrombosis Induction. Quantification of the thrombosis assay. (**A**) Ratio of vessels exhibiting thrombus formation, (**B**) average time required for initiation of thrombus formation, and (**C**) ratio of vessels in which flow was completely stopped due to thrombus formation in control mice (n = 8) and mice infused with hyaluronidase and heparinase (n = 5); means with 95% confidence intervals. * *p* < 0.05 and ns = nonsignificant.

The fluorescent excitation of Rose Bengal was used to stimulate thrombosis. Selected images of thrombus formation in a control mouse and a mouse that had been infused with the GAG-degrading enzymes are shown in Figure 6, at times of approximately 1, 5, 9, and 13 min following the infusion of the Rose Bengal dye. We found a significant increase in the ratio of retinal vessels in which thrombi formed when the mice were infused with heparanase/hyaluronidase than with the vehicle (Figure 7A; *p* < 0.05), with the thrombi tending to form more quickly with the prior degradation of GAGs (Figure 7B; *p* = 0.09). Over the 16 min observation period, more than twice as many retinal arterioles and venules completely stopped flowing due to thrombus formation in the mice given heparanase/hyaluronidase than in the controls (Figure 7C; *p* < 0.05).

## 4. Discussion

To our knowledge, this is the first study of the effect of the loss of endothelial glycocalyx on retinal hemodynamics in vivo. We observed a significant increase in arteriolar and venular diameters with the acute enzymatic degradation of the EG, with a more pronounced effect on the retinal venules. In addition, our findings indicate a significant decrease in venular shear rate with the infusion of hyaluronidase and heparinase. We further observed that the degradation of glycosaminoglycans causes a significant increase in total retinal blood flow and microhematocrit and an increased propensity for thrombus formation.

Previously, using a two-dye technique in the retinas of C57BL/6J mice, our lab measured arteriolar and venular glycocalyx thicknesses of 1.64 μm and 1.28 μm, respectively. In these measurements, the arteriolar and venular diameters filled with high molecular weight rhodamine dextran (155,000 MW, excluded from the EG) and low molecular weight sodium fluorescein (376 MW, glycocalyx permeable) were recorded using intravital microscopy. The difference between the two diameters (divided by 2) was calculated as the EG thickness [17]. In the current study, we observed a 4 μm increase in the arteriolar diameter with the enzymatic removal of EG, which could be consistent with a ~2 μm glycocalyx thickness. Therefore, the increase in arteriolar diameter following EG loss could be due merely to the reduction in the glycocalyx thickness, yielding more availability of the lumen for FITC–dextran filling. Interestingly, we observed a ~10 μm increase in the venular diameter following GAG degradation, indicating the involvement of additional factor(s)/mechanism(s) in the venular diameter change. In addition to the expanded lumen area due to glycocalyx loss, two other mechanisms of increased diameter can be discussed, i.e., shear-mediated dilation and pressure-induced vasoexpansion. The first of these two mechanisms, shear-mediated dilation, would be more likely to be a factor on the arteriolar side that contains vascular smooth muscle than on the venular side, which is, to the best of our knowledge, primarily devoid of smooth muscle. Therefore, pressure-induced vasoexpansion is the more likely explanation for the larger extent of diameter change in the venules compared to the arterioles; that is, pressure losses through the retinal circulation could be reduced following the loss of glycocalyx, which could result in increased pressure in the downstream venules.

The shear rate itself decreased in the venules with the loss of GAGs, but the microhematocrit (and therefore viscosity) significantly increased, and therefore, the directional change in shear stress (=viscosity × shear rate) is not completely straightforward. The same is true when considering one of the explanations for the increase in venular diameter with the loss of GAGs (pressure-induced vasoexpansion), where it can be noted that a decrease in resistance through the arterioles and capillaries could result in pressure-induced venular expansion, but again, the degree to which resistance is decreased (due to arteriolar/capillary loss of glycocalyx), could be offset somewhat by the potential increase in viscosity (microhematocrit). Nevertheless, given the overall increase in retinal blood flow with the loss of GAGs, it is more likely that resistance is decreasing. In our analyses, we made the assumption that average RBC velocity represented average blood velocity as a whole. However, it should be acknowledged that RBC velocity typically exceeds plasma velocity (especially in microvessels), with RBC concentrations higher toward the centerline than nearer the vessel wall, considering the parabolic profile of faster centerline velocity [30,31]. The exclusion of RBCs from the vessel wall is in part influenced by the endothelial glycocalyx matrix, with endothelial matrices also capable of altering the shear profiles [32]. Therefore, the velocities and shear rates that we present are approximations and can be influenced by our experimental protocol in which glycocalyx-degrading enzymes were infused.

Previously, a 14–21% decrease in flow resistance was observed with heparinase infusion in the arterioles of rat mesentery [8]; however, this study did not measure the diameter or blood flow change following EG degradation. Earlier simulation studies predicted that the presence of the EG provides resistance to blood flow and maintains the homogeneity of blood flow in the microvasculature [13,33]. The resistance generated by the EG is important, particularly at vessel bifurcations, because changes in diameter, resistance, and blood viscosity in the microvessels at bifurcations can cause inconsistencies in blood distribution [34]. The physical hindrance to flow that the glycocalyx provides likely contributes to vascular resistance, but the glycocalyx thickness can vary depending on the type and size of the vessel [1], and therefore it is of importance to determine whether the amount of resistance provided by the EG also varies with the size of the vessel.

We observed a significant increase in the number of RBCs per field of view with the loss of the EG. This is consistent with previous studies reporting an increase in capillary hematocrit after the degradation of the EG using various interventions, including heparinase or hyaluronidase [10,11,12]. Due to the presence of sulfated molecules, the EG is negatively charged and provides a physical hindrance; therefore, it repels blood cells, including RBCs [35], excluding them from the endothelial cell surface. If RBC flux remains constant, an increase in functional vessel diameter can cause an increase in vessel hematocrit. The vessel hematocrit can also be increased with substantial capillary fluid filtration or by reduced RBC velocity compared to other components in the blood [12]. We observed no significant changes in RBC velocity with the removal of EG. We previously observed an increase in retinal vascular leakage with hyaluronidase infusion [17], although the extent of the changes in fluid filtration with the loss of retinal glycocalyx remains unknown. In addition, the glycocalyx has been shown to decrease the impact of capillary irregularities on circulating RBCs [36] and promote higher deformability, which promotes the elongation of RBCs and longitudinal passage that can increase nutrient exchange in the capillary network [37]. A study using mathematical modeling projected that interactions between the glycocalyx on RBCs and the glycocalyx on endothelium are required for the gliding–hovering and rotation of RBCs passing through vessels, with rotational and hovering motions of RBCs being lost when the glycocalyx is degraded [38].

As with the human retina, the mouse retinal circulation is arranged in three general layers: the superficial layer closest to the inner side of the retina, the intermediate capillary layer, and the deep capillary layer. One relatively unique feature of retinal circulation is the hub-type radial arrangement of arteries and veins branching out of and into the central retinal artery and vein. A previous hemodynamic analysis of the mouse retinal circulation [39] reported that capillary velocity and microhematocrit are higher in the mouse retina than in other capillary beds. We have reported previously that the shunting of flow away from the deep retinal capillary layer, reducing the path length of blood flow, results in considerably faster transit times [24]. However, in this study, we observed no significant changes in velocities or mean circulation times with the acute loss of GAGs, suggesting no changes in the average path length. MCT can also be predicted by arteriolar diameters (D_A_ and D_V_) and flows using the expression (D_A_^2^ + D_V_^2^)/Flow Index [27,40]. We observed a significant increase in both diameters and flow with the degradation of the EG; an increase in both parameters (numerator, denominator) is consistent with minimal changes in the calculated MCT.

Lastly, the infusion of hyaluronidase and heparinase led to a significant reduction in venular shear rate. A reduced shear rate can cause increased rolling and adherence of the leukocytes, which further provides a platform for platelets to bind to the vasculature [41,42]. Previously, an increase in platelet–endothelial and leukocyte–endothelial adhesion has been reported with EG loss [43,44,45]. Together, the loss of the EG and a reduction in venular shear rates can cause pro-thrombotic conditions, also evident in our study, and may contribute to the progression of microvascular pathologies in diseases such as diabetic retinopathy. Previous studies from our lab and others have suggested a reduction in shear rate and EG thickness in the diabetic retina [10,15,16,25,46].

In conclusion, the loss of the endothelial glycocalyx can cause hemodynamic changes in the retinal microvasculature, including increases in lumen diameter, total blood flow, and microhematocrit. The increase in microhematocrit and retinal diameters have opposing influences on blood flow resistance. Given the increase in retinal blood flow, we can ascertain that the increase in diameter had a larger influence on resistance than microhematocrit. Furthermore, loss of the retinal EG causes a significant reduction in venular shear rates and an increased propensity for thrombus formation, which can contribute to the progression of microvascular diseases such as diabetic retinopathy. Although it is apparent from our study that the loss of EG regulates retinal hemodynamics and inflammation in the retina, 30 min of degradation is unlikely to trigger all the microvascular responses in chronic diseases such as diabetes; therefore, future studies are required to elucidate how the chronic loss of EG can cause or contribute to microvascular retinopathy.
